# High depression symptomatology and mental pain characterize suicidal psychiatric patients

**DOI:** 10.1192/j.eurpsy.2022.2312

**Published:** 2022-08-31

**Authors:** Maurizio Pompili, Marco Innamorati, Denise Erbuto, Mario Luciano, Gaia Sampogna, Giovanni Abbate-Daga, Stefano Barlati, Claudia Carmassi, Giovanni Castellini, Pasquale De Fazio, Giorgio Di Lorenzo, Marco Di Nicola, Silvia Ferrari, Arianna Goracci, Carla Gramaglia, Giovanni Martinotti, Maria Giulia Nanni, Massimo Pasquini, Federica Pinna, Nicola Poloni, Gianluca Serafini, Maria Signorelli, Alfonso Tortorella, Antonio Ventriglio, Umberto Volpe, Andrea Fiorillo

**Affiliations:** 1 Department of Neurosciences, Mental Health, and Sensory Organs, Faculty of Medicine and Psychology, Suicide Prevention Centre, Sant’Andrea Hospital, Sapienza University of Rome, Rome, Italy; 2 Department of Human Sciences, European University of Rome, Rome, Italy; 3 Department of Psychiatry, University of Campania “L. Vanvitelli”, Naples, Italy; 4 Eating Disorders Center, Department of Neuroscience, University of Turin, Turin, Italy; 5 Department of Clinical and Experimental Sciences, University of Brescia, Brescia, Italy; 6 Department of Mental Health and Addiction Services, ASST Spedali Civili of Brescia, Brescia, Italy; 7 Psychiatric Clinic, Department of Clinical and Experimental Medicine, University of Pisa, Pisa, Italy; 8 Psychiatry Unit, Department of Health Sciences, University of Florence, Florence, Italy; 9 Psychiatric Unit, Department of Health Sciences, University Magna Graecia, Catanzaro, Italy; 10 Department of Systems Medicine, University of Rome Tor Vergata, Rome, Italy; 11 Department of Neuroscience, Section of Psychiatry, Università Cattolica del Sacro Cuore, Rome, Italy; 12 Department of Psychiatry, Fondazione Policlinico Universitario Agostino Gemelli IRCCS, Rome, Italy; 13 Department of Biomedical, Metabolic and Neural Sciences, University of Modena and Reggio Emilia, Modena, Italy; 14 Dipartimento ad Attività Integrata di Salute Mentale e Dipendenze Patologiche, AUSL – IRCCS Reggio Emilia, Reggio Emilia, Italy; 15 Department of Molecular Medicine, University of Siena, Siena, Italy; 16 Department of Translational Medicine, Università del Piemonte Orientale, Novara, Italy; 17 Psychiatry Division, Azienda Ospedaliero Universitaria Maggiore della Carità, Novara, Italy; 18 Department of Neuroscience, Imaging and Clinical Sciences, University “G. D’Annunzio” of Chieti-Pescara, Chieti, Italy; 19 Department of Neuroscience and Rehabilitation, Institute of Psychiatry, University of Ferrara, Ferrara, Italy; 20 Department of Human Neurosciences, Sapienza University of Rome, Rome, Italy; 21 Section of Psychiatry, Department of Medical Sciences and Public Health, University of Cagliari, Cagliari, Italy; 22 Division of Psychiatry, Department of Medicine and Surgery, University of Insubria, Varese, Italy; 23 Department of Neuroscience, Rehabilitation, Ophthalmology, Genetics, and Maternal and Child Health, Psychiatry Section, University of Genoa, IRCCS San Martino, Genoa, Italy; 24 Department of Clinical and Experimental Medicine, AOU Policlinico Hospital, University of Catania, Catania, Italy; 25 Department of Psychiatry, University of Perugia, Perugia, Italy; 26 Department of Clinical and Experimental Medicine, University of Foggia, Foggia, Italy; 27 Unit of Clinical Psychiatry, Department of Neurosciences/DIMSC, School of Medicine and Surgery, Polytechnic University of Marche, Ancona, Italy

**Keywords:** Depression symptomatology, hopelessness, mental pain, mental pain, psychopharmacological medications, suicide behaviors and ideation

## Abstract

**Background:**

Symptoms of depression are transdiagnostic heterogenous features frequently assessed in psychiatric disorders, that impact the response to first-line treatment and are associated with higher suicide risk. This study assessed whether severe mental pain could characterize a specific phenotype of severely depressed high-risk psychiatric patients. We also aimed to analyze differences in treatments administered.

**Methods:**

2,297 adult patients (1,404 females and 893 males; mean age = 43.25 years, SD = 15.15) treated in several Italian psychiatric departments. Patients were assessed for psychiatric diagnoses, mental pain, symptoms of depression, hopelessness, and suicide risk.

**Results:**

More than 23% of the patients reported high depression symptomatology and high mental pain (HI DEP/HI PAIN). Compared to patients with lower symptoms of depression, HI DEP/HI PAIN is more frequent among females admitted to an inpatient department and is associated with higher hopelessness and suicide risk. In addition, HI DEP/HI PAIN (compared to both patients with lower symptoms of depression and patients with higher symptoms of depression but lower mental pain) were more frequently diagnosed in patients with personality disorders and had different treatments.

**Conclusions:**

Patients reporting severe symptoms of depression and high mental pain presented a mixture of particular dangerousness (high trait hopelessness and the presence of suicide ideation with more frequency and less controllability and previous suicide behaviors). The presence of severe mental pain may act synergically in expressing a clinical phenotype that is likewise treated with a more complex therapeutic regime than that administered to those experiencing symptoms of depression without mental pain.

## Introduction

Depressive symptomatology is a transdiagnostic feature frequently comorbid in psychiatric disorders, with highly heterogeneous symptoms, typically physical and emotional-cognitive symptoms encompassing feelings of worthlessness, guilt, shame, lowered self-esteem, and hopelessness. Heterogeneity and comorbidity of depression symptoms impact the response to first-line treatment [1–4], contributing to higher suicide risk. Some authors consider mental pain a part of depression, although doubts still exist about a clear distinction between major depression and mental pain [5, 6]. To date, symptoms of depression and mental pain have been indicated as explanatory constructs that, at least in part, convincingly pave the way to the wish to die [6].

Scholars have reported that mental pain is higher in suicide attempters than nonattempters and suicide ideators than nonideators [7–9]. Furthermore, emotional and cognitive symptoms of depression notably associated with suffering may resemble the experience of mental pain. However, mental pain can be distinguished from symptoms of depression for peculiar features (especially when negative emotions such as fear, shame, guilt, and narcissistic wounds are part of the suffering, together with a range of subjective experiences characterized as an awareness of negative changes in the self and in its functions accompanied by negative feelings [10]), and overall characterizes suicide risk when an individual’s overwhelming subjective experience overcomes one’s threshold for enduring or tolerating pain [11, 12]. Depressive symptoms encompass diagnoses and may play a central role in major psychiatric disorders to recognize more vulnerable subpopulations [13–15].

Additionally, the severity of depressive symptoms in major depressive disorder (MDD) was reported to be associated with an increased risk of an MDD-related hospital encounter in the short term [16]. A recent study showed that patients with MDD and suicidal ideation (vs. patients without suicidal ideation and the general population) had significantly worse quality of life, high healthcare resource use, and greater productivity loss and activity impediment [17]. Therefore, it would appear that patients of this population represent a subgroup of depressive patients with a more significant burden. Although symptoms of depression may identify more vulnerable patients across the psychiatric diagnoses for suicide risk assessment, the inclusion of mental pain evaluation may represent an innovative way to help clinicians better manage these patients. A study investigating the functional neuroanatomy of depressed patients with mental pain also suggested differences in depressed patients with lower mental pain [18]. In this study, mental pain was associated with change in brain areas involved in emotional processing [18].

## Aims

The present study, therefore, aimed to assess whether severe mental pain could characterize a specific phenotype of severely depressed high-risk psychiatric patients. We hypothesized that regardless of the diagnoses, patients with current self-reported symptoms of severe depression and severe mental pain are at higher suicide risk and are more prone to trait hopelessness when compared with psychiatric patients with severe symptoms of depression but lower mental pain. Since pharmacological treatment represents a major therapeutic intervention for properly managing given diagnoses and treating specific symptoms, including the manifestation of depression, we aimed to analyze treatments administered to severely depressed patients with severe psychological pain to assess whether these differed from depressed patients with lower psychological pain.

## Methods

A multicenter observational study was a joint project with numerous mixed Italian academic and clinical settings (represented by the authors’ affiliations). We reported complete information in a previous article [19]. Briefly, the project was first submitted to the Internal Review Board of Sant’Andrea Hospital (RIF.CE: 4646_2017) as part of the Sapienza University of Rome, the study’s coordinator. The protocol was then submitted to the internal review boards of the participating centers by local investigators.

The study was conducted ethically following the World Medical Association Declaration of Helsinki. Patients participated voluntarily and provided written informed consent following review and approval of each participating center’s research ethics review board, ensuring that data would be reported anonymously and in aggregate form.

### Participants

Participants were 2,297 adult patients (1,635 outpatients and 662 inpatients; 1,404 females and 893 males; mean age = 43.25 years, SD = 15.15, age range = 18–95 years) treated in several psychiatric inpatient and outpatient departments.

Patients were assessed for psychiatric diagnoses according to the Diagnostic and Statistical Manual of Mental Disorders (DSM-5) criteria, the Structured Clinical Interview 5th Edition (SCID—DSM-5) [20], via psychiatric interviews and the administration of psychometric instruments. The inclusion criterion was to be between 18 and 65 years of age (subjects over 65 were included only if physically fit; that is, they lacked medical comorbidities that reduced quality of life, caused impairments, etc.). In addition, exclusion criteria were being unwilling to participate or denying informed consent and having neurological diseases (e.g., dementia, Parkinson’s disease, and epilepsy), cognitive impairments, and language difficulties.

A web-based system with codes and anonymous labels was devised for this study. Each center entered all data via a guided digital procedure to minimize data entry errors.

### Measures

All the patients were administered a comprehensive battery of psychological tests and semi-structured interviews, including the Physical and Psychological Pain Scale (PPPS) [21], the Beck Depression Inventory-II (BDI-II) [22], the Beck Hopelessness Scale (BHS) [23], and the Columbia Suicide Severity Rating Scale (C-SSRS) [24]. For a proper definition of suicide attempt, we referred to [25, 26]. Type II suicide attempts are described as self-destructive acts with some degree of intent to end one’s life and some identifiable injuries. Suicide attempts were considered recent when they had occurred in the past 3 months, following the C-SSRS assessment, as this was the main instrument used for this purpose. It is a rating scale evaluating suicidal ideation in individuals aged 12 years and older [24].

#### Columbia Suicide Severity Rating Scale

A rating scale evaluating suicidal ideation in individuals aged 12 years and older [24]. The C-SSRS rates an individual’s degree of suicidal ideation on a scale from “wish to be dead” to “active suicidal ideation with a specific plan and intent.” The C-SSRS begins with two items assessing the respondent’s wish to be dead (e.g., “I wish I were dead”) and nonspecific active suicidal thoughts (e.g., “I have thought about killing myself”). If the participant responds positively to these items, then three additional items are used to assess: active suicidal ideation either with any method but no plan or intent to act; active suicidal ideation with some intent to act but no plan; and active suicidal ideation with a specific plan and intent. According to the protocol of this instrument, we used past month ratings for all analyses involving suicidal ideation and intent, whereas past 3 month ratings for those involving suicidal behavior.

#### Physical and Psychological Pain Scale

A self-administered questionnaire that evaluates the intensity of physical and mental pain on a dimensional scale from 0 (*none*) to 10 (*maximum possible pain*) [21]: the current intensity of pain, the usual pain during the last 15 days, and the maximum pain always experienced in relation to the last 15 days (PPPS worst) [21]. Cronbach’s alpha values in the present sample were 0.91 and 0.92, respectively, for physical and mental pain dimensions.

#### Beck Depression Inventory-II

A 21-item self-report instrument evaluating the presence/severity of depressive symptoms during the previous 14 days [22]. A single item assesses suicide ideation (item no. 9) and has been considered an efficient screening tool for suicide risk in clinical settings [27]. A sum score of all items, except for the item assessing suicide ideation, was calculated as an index of depression severity. For this study, we considered a cut-off score of 20 for screening patients with current moderate to severe self-reported depression. A score of 2 (“I would like to kill myself,” “I would kill myself if I had the chance”) or higher on the item assessing suicide ideation was used as an index of higher current suicide risk (i.e., indicated as “Serious suicide ideation”). Score of 0 on item 9 indicated that suicide risk in the past 14 days was weak or nonpresent, score of 1 indicated the presence of death wishes. Cronbach’s alpha in the present sample was 0.94.

#### Beck Hopelessness Scale

A 20-item self-report measure of trait hopelessness about the future [28, 29]. Higher scores indicate more severe hopelessness. Several international studies reported good psychometric properties of the BHS and suggested satisfactory ability in predicting subsequent suicide behavior, and general health and social functioning [30–34]. A score of 9 or higher could detect patients at risk for suicide [31, 35]. In Italy, validation studies have been conducted on samples of medical patients, university students, and psychiatric inpatients and have demonstrated satisfactory psychometric properties [30]. Cronbach’s alpha in the present sample was 0.89.

### Statistical analyses

Patients were included in three groups according to their self-reported depression and worst psychological pain levels in the past two weeks. Patients with BDI-2 scores ≥20 and psychological pain scores in the upper quartile (≥75th percentile) were included in the high depression symptomatology and high psychological pain (HI DEP/HI PAIN) group. Patients with BDI-2 scores ≥20 and psychological pain scores in the lower quartiles (<75th percentile) were included in the HI DEP/LO PAIN group. Patients with BDI-2 scores <20 were included in the low depression symptomatology (LO DEP) group. Considering the high number of categories, DSM-5 diagnoses were included in two separate variables, a variable for personality disorders (with two categories for borderline personality disorder and other personality disorders) and one other variable for all diagnoses. In the DSM-5 diagnosis variable, all patients included in the category none had a personality disorders or substance abuse.

A series of ANOVAs and chi-squared tests were used to evaluate bivariate differences among groups. Bonferroni correction was used to correct for multitesting, and measures of effect sizes were reported. Partial eta squared of 0.01 indicates a small effect, ≥ 0.06 medium effect, ≥ 0.14 large effect. Cramer’s *v* ≤ 0.2 indicates a small effect, *v* ≤ 0.6 indicates a medium effect, and *v* > 0.6 indicates a large effect [36].

Variables significant in the bivariate analyses after correction for multitesting (except for DSM-5 diagnoses) were included in multinomial regression models with groups as a criterion. Odds ratio (OR) and their 95% confidence intervals (CI) are reported as measures of association. In addition, DSM-5 diagnoses and treatments were included in a separate log-linear model to analyze further the links between these variables and the presence of mental pain.

## Results

### Factors associated with HI DEP/HI PAIN

In the past two weeks, 535 patients reported HI DEP/HI PAIN, 550 reported high depression symptomatology (BDI ≥ 20), but low psychological pain (below the 75th percentile) (HI DEP/LO PAIN), and 1,188 reported LO DEP.

In Table 1, sociodemographic and clinical characteristics of the groups are reported. After controlling for multitesting, groups differed according to sex, DSM-5 diagnosis, presence of personality disorders (PDs), number of inpatients, BHS scores, lifetime suicide attempts, BDI-2 suicide risk, recent nonsuicidal self-harm, and for all treatments. Post hoc comparisons were significant (*p* < 0.05) with HI DEP/HI PAIN > HI DEP/LO PAIN > LO DEP for the presence of females, PDs, BHS scores, lifetime suicide attempts, BDI-2 suicide risk, recent nonsuicidal self-harm, and antipsychotic and anxiolytic treatments. Patients with HI DEP/HI PAIN had also been prescribed mood stabilizers more frequently and were more often inpatients than the other two groups, while the HI DEP/HI PAIN and HI DEP/LO PAIN patients had more frequently been prescribed antidepressants than the LO DEP group. Finally, HI DEP/HI PAIN and HI DEP/LO PAIN patients differed from LO DEP regarding DSM-5 diagnoses. LO DEP patients (compared to the HI DEP groups) more frequently had diagnoses of schizophrenia and other psychoses (17.2% vs. 11.5% vs. 11.8%, respectively for LO DEP, HI DEP/LO PAIN, HI DEP/HI PAIN) or other specified DSM-5 diagnoses (8.9% vs. 3.8% vs. 3.7%, respectively for LO DEP, HI DEP/LO PAIN, HI DEP/HI PAIN), and less frequently had diagnoses of MDD (21.5% vs. 31.5% vs. 34.8%, respectively for LO DEP, HI DEP/LO PAIN, HI DEP/HI PAIN) and eating disorders (8.1% vs. 11.7% vs. 12.9%, respectively for LO DEP, HI DEP/LO PAIN, HI DEP/HI PAIN).

**Table 1.** Bivariate analyses.

	Whole sample *N* = 2,297								
Variables	*N*	%	1. Low depression *N* = 1,188	2. High depression/Low psychological pain *N* = 550	3. High depression/High psychological pain *N* = 535	Test	Significance	Post hoc comparisons: *p* < 0.05	Effect size
Sex						*χ* ^2^_2_ = 44.91	<0.001	All	*v* = 0.14
Males	893	38.9%	45.3%	35.6%	29.0%				
Females	1,404	61.1%	54.7%	64.4%	71.0%				
Age – M(SD)	43.25	(15.15)	43.91(15.06)	43.38(14.87)	41.79(15.50)	*F*_2;2,229_ = 3.54	0.03	—	Partial eta^2^ = 0.003
Marital status						*χ* ^2^_4_ = 14.05	0.007	—	*v* = 0.06
Married	757	33.0%	35.2%	34.9%	27.0%				
Divorced or widowed	380	16.5%	15.4%	15.9%	20.2%				
Single	1,137	49.5%	49.3%	49.2%	52.8%				
Job						*χ* ^2^_4_ = 13.31	0.01	—	*v* = 0.06
Employed	1,133	49.3%	52.9%	49.2%	44.9%				
Unemployed	799	34.8%	32.3%	37.6%	40.7%				
Other	326	14.2%	14.8%	13.2%	14.5%				
School attainment						*χ* ^2^_4_ = 15.65	0.004	—	*v* = 0.06
≤8 years	755	32.9%	30.1%	36.6%	36.5%				
= 13 years	1,128	49.1%	51.2%	45.8%	50.3%				
≥16 years	385	16.8%	18.6%	17.6%	13.2%				
Living condition						*χ* ^2^_4_ = 4.90	0.30	—	*v* = 0.03
Alone	458	19.9%	19.9%	17.7%	22.7%				
Family or friends	1,513	65.9%	66.6%	67.3%	64.8%				
Other	308	13.4%	13.4%	15.0%	12.5%				
Diagnosis						*χ* ^2^_14_ = 85.99	<0.001	12,13	*v* = 0.14
None	194	8.4%	8.6%	7.9%	9.0%				
Schizophrenia and other psychoses	330	14.4%	17.2%	11.5%	11.8%				
Bipolar disorder	510	22.2%	23.1%	22.2%	19.7%				
Major depressive disorder	616	26.8%	21.5%	31.5%	34.8%				
Anxiety disorder	153	6.7%	7.9%	6.6%	3.9%				
Stress and trauma correlated disorders	104	4.5%	4.6%	4.8%	4.1%				
Eating disorders	231	10.1%	8.1%	11.7%	12.9%				
Other	149	6.5%	8.9%	3.8%	3.7%				
Personality disorders						*χ* ^2^_4_ = 78.19	<0.001	All	*v* = 0.14
None	1,688	73.5%	83.5%	78.3%	64.6%				
Borderline personality disorder	176	7.7%	5.2%	7.6%	16.0%				
Other personality disorders	299	13.0%	11.3%	14.1%	19.4%				
Substance use disorder	197	9.1%	11.0%	7.3%	7.0%	*χ* ^2^_2_ = 9.65	0.008	—	*v* = 0.07
Inpatients	662	28.8%	24.8%	27.7%	47.7%	*χ* ^2^_2_ = 83.06	<0.001	13,23	*v* = 0.20
BDI-2 depression^a^	20.18	(13.66)	9.31(5.77)	28.62(7.43)	35.22(9.89)	—	—		—
Olie Worst recent mental pain—M/75^b^ Percentile (SD)	6.11/9	(3.41)	4.42(3.39)	6.21(2.11)	9.69(.47)	—	—		—
BHS − M(SD)	9.08	(5.53)	6.29(4.48)	11.16(4.78)	13.00(4.98)	*F*_2;2,229_ = 452.52	<0.001	All	Partial eta^2^ = 0.29
Lifetime suicide attempts	852	37.1%	28.2%	40.7%	53.3%	*χ* ^2^_2_ = 103.35	<0.001	All	*v* = 0.21
BDI-2 suicide risk (past 14 days)						*χ* ^2^_4_ = 511.11	<0.001	All	*v* = 0.34
None or weak	1,929	84.0%	98.5%	82.7%	56.3%				
Death wishes	271	11.8%	1.4%	14.0%	32.4%				
Serious suicide ideation	80	3.5%	0.1%	3.3%	11.3%				
Recent nonsuicidal self-harm	307	13.4%	8.3%	14.2%	23.8%	*χ* ^2^_2_ = 76.46	<0.001	All	*v* = 0.18
Treatments									
Antipsychotics	1,050	45.7%	49.1%	55.2%	63.9%	*χ* ^2^_2_ = 27.67	<0.001	All	*v* = 0.12
Antidepressants	1,069	46.5%	47.1%	61.6%	63.9%	*χ* ^2^_2_ = 47.71	<0.001	12,13	*v* = 0.16
Mood stabilizers	847	36.9%	40.6%	45.3%	52.9%	*χ* ^2^_2_ = 18.48	<0.001	13,23	*v* = 0.10
Anxiolytics	1,053	45.8%	47.2%	58.1%	68.1%	*χ* ^2^_2_ = 56.50	<0.001	All	*v* = 0.17

*Note:* This score was used to categorize patients (i.e., patients with High depression/Low psychological pain have Olie Worst recent mental pain scores < 75° percentile, while High depression/High psychological pain patients have Olie Worst recent mental pain scores ≥ 75° percentile); Correction for multitesting: *p* = 0.05/17 = 0.003.

Abbreviations: BDI-2, Beck Depression Inventory-2; BHS, Beck Hopelessness Scale.

a BDI-2 sum score without item 9 score.

b Olie Worst recent mental pain scores are reported as mean ± standard deviation as for other variables, but also the 75° percentile score is reported.

All variables (excluding DSM-5 diagnoses) were included in a multivariate model with groups as the criterion (Table 2). When controlling for other variables, groups differed for all the variables (excluding lifetime suicide attempts, recent nonsuicidal self-harm, and treatments with antipsychotics and mood stabilizers). Compared to LO DEP patients, both HI DEP/HI PAIN and HI DEP/LO PAIN patients were more frequently females, had higher BHS scores, had higher BDI-2 suicide risk, and were treated more frequently with antidepressants and anxiolytics. HI DEP/HI PAIN patients (compared to LO DEP) were also more frequently diagnosed with PDs and admitted to an inpatient department.

**Table 2.** Multivariate model.

	Model			HI DEP/LO PAIN versus LO DEP			HI DEP/HI PAIN versus LO DEP		
Variables	*χ* ^2^	*df*	Significance	Odds ratio	95% CI—lower limit	95% CI—upper limit	Odds ratio	95% CI—lower limit	95% CI—upper limit
BHS	293.71	2	<0.001	1.21	1.18	1.24	1.27	1.22	1.31
Males	29.04	2	<0.001	0.68	0.52	0.89	0.41	0.29	0.57
Personality disorders	8.86	2	0.01	1.23	0.87	1.74	1.77	1.21	2.59
BDI-2 suicide risk (past 14 days)	180.32	4	<0.001						
Death wishes				8.26	4.26	15.99	23.75	12.32	45.78
Serious suicide ideation				20.24	2.52	162.50	83.72	10.73	653.02
Lifetime suicide attempts	1.05	2	0.59	0.86	0.64	1.16	0.94	0.67	1.34
Recent nonsuicidal self-harm	0.48	2	0.79	0.88	0.58	1.32	0.97	0.62	1.52
Antipsychotics	3.70	2	0.16	1.24	0.94	1.65	1.35	0.97	1.89
Antidepressants	10.22	2	0.01	1.47	1.13	1.91	1.52	1.10	2.09
Mood stabilizers	3.23	2	0.20	1.12	0.85	1.48	1.35	0.97	1.87
Anxiolytics	11.33	2	0.01	1.54	1.17	2.02	1.52	1.10	2.11
Inpatients	26.58	2	<0.001	1.07	0.79	1.46	2.28	1.61	3.23

*Note:* Model fit: −2LL=2,384.41, *χ* ^2^_24_=847.95, *p* < 0.001; Nagelkerke *R* ^2^ = 0.45.

Abbreviations: BDI-2, Beck Depression Inventory-2; BHS, Beck Hopelessness Scale.

To further differentiate HI DEP/HI PAIN from HI DEP/LO PAIN patients, diagnoses and treatments were included in a log-linear model with groups as the criterion (HI DEP/HI PAIN vs. HI DEP/LO PAIN) (not reported in the tables). The model fitted the data well (Pearson *χ* ^2^_181_ = 137.36, *p* = 0.99. HI DEP/HI PAIN and HI DEP/LO PAIN did not differ for DSM-5 diagnoses (*p* between 0.053 for other specified diagnoses and 0.93 for MDD) and the frequency of antidepressant treatments (*p* = 0.21). HI DEP/HI PAIN (compared to HI DEP/LO PAIN) were more frequently diagnosed with personality disorders (OR = 1.91, *p* < 0.001), and they were more frequently treated with antipsychotics (OR = 1.58, *p* < 0.01), mood stabilizers (OR = 1.53, *p* < 0.01), and anxiolytics (OR = 1.34, *p* < 0.05).

### Differences among groups on the C-SSRS regarding last month’s suicide ideation

Groups were compared for last month’s suicide ideation according to the C-SSRS (Table 3). DEP/PAIN groups significantly (*p* < 0.05) differed in the presence of suicide ideation and characteristics of the ideation (frequency, duration, controllability, the presence of deterrents, and pain as reason for suicide ideation). Post hoc comparisons were significant (*p* < 0.05) for all the variables. HI DEP/HI PAIN patients differed from the LO DEP group and from HI DEP/LO PAIN patients for the presence of more severe suicide ideation and for the existence of higher frequency, more duration, less controllability, fewer deterrents, and for having higher pain as a reason for suicide ideation than other groups.

**Table 3.** Columbia Suicide Severity Rating Scale (C-SSRS) last month suicide ideation.

	Whole sample *N* = 2,297								
Variables	*N*	%	1. Low depression *N* = 1,188	2. High depression/Low psychological pain *N* = 550	3. High depression/High psychological pain *N* = 535	Test	Significance	Post hoc comparisons: *p* < 0.05	Effect size
Suicide ideation last month									
None	1,454	63.3%	82.8%	59.2%	25.8%	*χ* ^2^_10_ = 592.08	<0.001	All	*v* = 0.36
Wish to be dead	230	10.0%	6.1%	14.2%	14.0%				
Suicidal thoughts	94	4.1%	1.9%	5.6%	7.5%				
Suicidal thoughts with method (but without specific plan or intent to act)	131	5.7%	1.9%	7.1%	12.1%				
Suicidal intent (without specific plan)	130	5.7%	2.9%	6.2%	11.2%				
Suicidal intent with specific plan	255	11.1%	4.5%	7.7%	29.3%				
Frequency	0.95	(1.56)	0.37(1.02)	0.97(1.49)	2.17(1.86)	*F*_2;2,270_ = 316.51	<0.001	All	Partial eta^2^ = 0.22
Duration	0.79	(1.36)	0.31(.89)	0.76(1.22)	1.85(1.67)	*F*_2;2,270_ = 304.90	<0.001	All	Partial eta^2^ = 0.21
Controllability	0.81	(1.44)	0.31(.91)	0.84(1.39)	1.86(1.77)	*F*_2;2,270_ = 273.19	<0.001	All	Partial eta^2^ = 0.19
Deterrents	0.72	(1.34)	0.31(.96)	0.67(1.22)	1.67(1.68)	*F*_2;2,270_ = 227.45	<0.001	All	Partial eta^2^ = 0.17
Pain as reasons for suicide ideation	1.26	(1.95)	0.52(1.40)	1.38(2.00)	2.73(2.05)	*F*_2;2,270_ = 304.42	<0.001	All	Partial eta^2^ = 0.21

Variables characterizing the previous month’s suicide ideation were included as predictors in a multinomial regression model with DEP/PAIN groups as the criterion (not reported in a table). The model fitted the data well (−2LL = 1,237.42, *χ* ^2^_10_ = 568.92, *p* < 0.001; Nagelkerke *R* ^2^ = 0.26). All the variables were significantly associated with DEP/PAIN groups (*p* < 0.05). Compared to the LO DEP patients, HI DEP/LO PAIN patients reported significantly higher frequency (OR = 1.19, 95% CI = 1.01/1.41), lower controllability (OR = 1.19, 95% CI = 1.02/1.43), and pain more frequently as a motivation for suicide ideation (OR = 1.22, 95% CI = 1.10/1.35) but no differences related to duration (OR = 0.94, 95% CI = 0.76/1.16) and deterrents (OR = 0.87, 95% CI = 0.75/1.01). Compared to the LO DEP group, HI DEP/HI PAIN patients reported significantly higher frequency (OR = 1.30, 95% CI = 1.11/1.51), lower controllability (OR = 1.20, 95% CI = 1.03/1.39), and pain more frequently as a motivation for suicide ideation (OR = 1.30, 95% CI = 1.18/1.43) but no differences regarding duration (OR = 1.16, 95% CI = 0.96/1.40) and deterrents (OR = 1.04, 95% CI = 0.91/1.19).

## Discussion

This study sought to characterize a mixed diagnoses group of depressed, suicidal versus nonsuicidal psychiatric inpatients according to the degree of mental pain. The study also explored the role of pharmacotherapy, specifically if patients received different therapeutic regimes according to the degree of mental pain assessed by clinicians. Overall, the comorbidity of depression and mental pain symptoms appeared deleterious for suicide risk. These patients differed in sociodemographic and clinical characteristics and presented a mixture of particular dangerousness. More than 23% of the sample reported HI DEP/HI PAIN. Thus, mental pain is highly prevalent in depressed psychiatric patients, especially in inpatient settings. More than 47% of our patients with HI DEP/HI PAIN were treated in inpatient wards. The presence of HI DEP/HI PAIN in hospitalized psychiatric patients could partly explain the association between suicide risk status and the utilization of psychiatric services. Of note is that the introduction of the assessment of mental pain could help in the complex issue of inpatient suicides. Findings have shown that suicide risk is particularly high during hospitalization and at discharge [37].

In Deisenhammer et al. [38] study, those who died by suicide during hospitalization more frequently had an increased history of attempted suicide; they less often reported future plans and more regularly showed no improvement in mood at discharge. The opportunity to identify the presence of mental pain comorbid with depressive symptoms may help recognize poor future expectations, which is a well-known proxy for completed suicide [39].

For example, around 79% of patients with HI DEP/HI PAIN in our sample also reported high trait hopelessness (BHS > 9), denoting severe pessimism, poor expectancies for future positive events, and poor future orientation. In our sample, patients with HI DEP/HI PAIN also showed a gender gap, with females more highly represented in this subpopulation of patients (>70%). Such findings may be relevant compared to a study investigating gender inequalities in inpatient suicide rates during psychiatric inpatient treatment and after discharge in 12 countries. The authors reported a similar gender gap in suicide risk, with females having higher suicide rates during and after hospitalization [40]. However, contrasting results have been reported, with a higher risk in male patients [41].

Therefore, the subpopulation of HI DEP/HI PAIN patients was a high-risk category. They reported a long-lasting, higher suicide risk than other patients. Current suicide ideation was reported with more frequency and less controllability, and lifetime suicide attempts were more frequent than other patients. According to Zou et al. [42], the higher risk of suicide in patients with severe mental pain could be explained by a specific component of mental pain, pain avoidance, and the behavioral tendency to escape from psychological pain through suicide [43].

Psychiatric diagnoses did not help differentiate this group from patients with HI DEP/LO PAIN, except that they more frequently had a personality disorder diagnosis (often a borderline personality disorder). Nevertheless, mental pain in depressed patients was associated with differences in the treatments administered. The presence of current severe mental pain was related to the more frequent prescribing of antipsychotics, mood stabilizers, and anxiolytics. It is reasonable to acknowledge that depressive symptoms and mental pain may act synergically in expressing a clinical phenotype that is likewise treated with a more complex therapeutic regime than in patients experiencing symptoms of depression without mental pain. We found that patients with HI DEP/HI PAIN (compared to HI DEP/LO PAIN) were more often treated with antipsychotics, mood stabilizers, and anxiolytics. It seems reasonable to assume that mental pain as a form of inner turmoil may require sedative, tranquilizing, and mood-modulation agents to relieve patients’ suffering [44–48].

Such possible implications of psychopharmacology in psychiatric patients experiencing mental pain have been reported as central to implementing patient-reported outcomes; any report comes directly from patients about how they function or feel concerning a health condition or therapy [49]. In addition, Fava et al. [49] pointed to the transdiagnostic features of mental pain, as such features are traceable to several psychiatric disorders, such as depression, anxiety, eating disorders, and borderline personality disorder.

Mental pain features are also crucial for the early detection of suicide risk [50]. In a previous article, we reported that recent suicide attempters (compared to nonattempters) had a more severe childhood physical, sexual, and emotional abuse history but not emotional and physical neglect (for this dimension, we have to consider that reliability was insufficient). Moderated mediation models indicated that childhood trauma was directly and indirectly associated with the presence of a recent suicide attempt. Mental pain (usual and worst mental pain in the last 15 days) partly mediated the relationship between childhood trauma and suicide status [19]. The present report adds consistent emphasis on the need to explore patients’ perspectives through an empathic understanding of their sufferance as part of the clinical picture [51–53]. Therefore, there is a need to bridge the gap in communication of human suffering and fine-tune the inner dialogue represented by painful thoughts of reflections and considerations of handling such pain. Individuals may give up on an extreme gesture that will abolish the unfortunate state. Reducing the flow of consciousness is the ultimate goal to achieve some relief. Pharmacological compounds that act to tranquilize patients may be helpful in the acute states most associated with suicide risk.

### Limitations

Although this is one of the most extensive investigations into the role of mental pain and depressive symptoms in explaining suicide risk among psychiatric patients, there are limitations. First, the many centers participating in this research may point to the heterogeneity of assessment and evaluation procedures through psychometric instruments. Second, patients had several psychiatric diagnoses, making it difficult to characterize single groups. Furthermore, some centers did not manage to recruit the number of patients set at the beginning of the study, thus influencing the heterogeneity and representativeness of the final sample. Finally, there are also limitations concerning the instruments used to assess the patients (such as C-SSRS that assessed suicidal intent in the past month, whereas recent suicide attempts were considered to be in the past three months, which could set the two measures at different times).

## Conclusions

The results presented in this article demonstrate that the combination of depressive symptoms and mental pain in psychiatric patients is a critical condition that increases suicide risk. Conversely, such risk was lower in patients who reported high rates of depressive symptoms but low rates of mental pain. Therefore, the former construct is a strategic perspective for the clinician for adding a complete understanding of patients’ mental status in their assessment and ultimately in delivering tailored treatments.

## Data Availability

The ethics committee did not grant permission to share study data with third parties or to upload data in anonymized form.
